# Retraction Note to: The role of seed appendage in improving the adaptation of a species in definite seasons: a case study of *Atriplex centralasiatica*

**DOI:** 10.1186/s12870-020-02482-5

**Published:** 2020-06-15

**Authors:** Zhaoren Wang, Yufei Zhao, Yuanyuan Zhang, Baoshan Zhao, Zhen’an Yang, Lijia Dong

**Affiliations:** 1 School of Life Sciences, Shaoxing University, Shaoxing, Zhejiang People’s Republic of China; 2 State Key Laboratory of Vegetation and Environmental Change, Institute of Botany, Chinese Academy of Sciences, Beijing, People’s Republic of China; 3 College of Life Science, China West Normal University, Nanchong, Sichuan People’s Republic of China; 4 College of life science, Shanxi Normal University, Linfen, Shanxi People’s Republic of China; 5 University of Chinese Academy of Sciences, Beijing, People’s Republic of China

**Retraction Note to: BMC Plant Biol (2019)19:538**

**https://doi.org/10.1186/s12870-019-2090-6**

The authors have retracted this article [1] because they did not have permission from the data owner to publish part of the data presented here.

All authors have responded to the correspondence and agree to this retraction.
